# Beyond Chemotherapy: Network Meta‐Analysis Reveals Optimal Neoadjuvant Strategies for Luminal Breast Cancer

**DOI:** 10.1002/cam4.71648

**Published:** 2026-02-13

**Authors:** Xinyu Li, Peijing Du, Tao Huang

**Affiliations:** ^1^ Department of Breast and Thyroid Surgery, Union Hospital, Tongji Medical College Huazhong University of Science and Technology Wuhan China; ^2^ Ningxia Medical University Yinchuan China

**Keywords:** breast cancer, endocrine therapy, hormone receptor positive, neoadjuvant therapy

## Abstract

**Background:**

Hormone receptor‐positive (HR+), HER2‐negative (HER2−) breast cancer represents the most common subtype. Given its distinct biology, neoadjuvant endocrine therapy (NET) offers comparable efficacy to neoadjuvant chemotherapy (NCT) with less toxicity. This systematic review and network meta‐analysis evaluates the evidence to guide clinical decision‐making for locally advanced or inoperable HR+/HER2− breast cancer.

**Methods:**

We analyzed phase II/III neoadjuvant clinical trials in HR+/HER2− breast cancer. Primary endpoints were overall response rate (ORR) by palpation and imaging. Secondary endpoints included breast‐conserving surgery (BCS) rates, pathological complete response (pCR), and safety. Treatment efficacy was ranked using surface under the cumulative ranking curve (SUCRA).

**Results:**

A total of 5181 patients across 21 trials were included in the study. CDK4/6 inhibitor + ET ranked highest for ORR by palpation (90.9%), and BCS (77.1%), followed by aromatase inhibitors (76.1% and 74.4%, respectively). For ORR by radiography, chemotherapy ranked first (87.6%) followed by the tyrosine kinase inhibitor (TKI) plus ET (76.7%). TKI + ET ranked first in pCR (79.6%), followed by chemotherapy (76.1%). Selective estrogen receptor degraders were the most tolerable, with the highest ranking in completion rate (84.1%) and fewer ≥ grade 3 adverse events (90.4%).

**Conclusions:**

NET is a viable alternative to NCT in HR+/HER2− patients. CDK4/6 + ET demonstrates superior tumor reduction and safety, potentially enabling postoperative therapy de‐escalation. These findings support NET as a strategic option for optimizing outcomes while minimizing toxicity.

## 1 Introduction

Hormone receptor‐positive (HR+) breast cancer, also known as the Luminal subtype, constitutes approximately 70% of all Breast Cancer (BC) [1]. It is characterized by the unique expression of estrogen and/or progesterone receptor. Distinct from other subtypes, HR+ breast cancer exhibits lower sensitivity to chemotherapy (CT) but responds effectively to hormonal agents [2]. For elderly patients with comorbidities or those who cannot tolerate CT, endocrine therapy (ET) offers an alternative treatment with fewer adverse effects and better adherence, without compromising therapeutic effect [3]. The fundamental pharmacological mechanism of ET involves disruption of ER signaling pathway which typically by interfering the binding of estrogen with ER (e.g., Tamoxifen), reducing estrogen production (e.g., Letrozole, Exemestane) and degradation of ER (e.g., fulvestrant) [4].

The emergence of CDK4/6 inhibitors has further enhanced the efficacy of endocrine therapy in HR+/HER2− patients. Clinical studies on advanced breast cancer (ABC) involving CDK4/6 inhibitors, such as Monarch‐3 [5], MONALEESA‐7 [6], and PALOMA‐2 [7], have demonstrated significant improvements in progression‐free survival (PFS) and overall survival (OS) compared to single‐agent hormone therapy. Additionally, the RIGHT Choice study showed comparable outcomes between CDK4/6 inhibitors combined with ET and traditional CT in aggressive HR+/HER2− ABC [8].

Neoadjuvant therapy is established as the standard regimen for high‐risk early breast cancer (EBC) and locally advanced breast cancer (LBC) [9]. The primary goal of preoperative therapy is to reduce tumor size, thereby optimizing cosmetic outcomes [10]. Moreover, comparing pre‐ and post‐surgical specimens allows for personalized systemic therapy based on individual biological features [11]. For HR+/HER2− LBC, whether receiving CT or ET, the absolute cases in terms of pathological complete response (pCR) are much lower than in other subtypes, whether treated with CT or ET [12]. However, previous studies reported no significant association between pCR and prognosis [13, 14]. Alternative endpoints, such as overall response rate (ORR), breast conservation rate (BCS), and PEPI score, have shown better indications of the therapeutic efficacy [15, 16, 17]. Previous studies comparing neoadjuvant endocrine therapy (NET) and neoadjuvant chemotherapy (NCT) have yielded inconsistent conclusions regarding the right choice of neoadjuvant treatment [18, 19]. Given the impressive ORR of CDK4/6i in treatment‐naïve ABC, research has shifted toward exploring CDK4/6 inhibitor‐based ET in the preoperative setting, challenging the traditional role of NCT [18]. However, the widespread adoption of NET is limited by its long treatment duration and uncertainties regarding therapeutic efficacy [17].

Here, we performed a comprehensive systemic review and network analysis of different neoadjuvant regimens for HR+/HER2− breast cancer. By which we aimed to find the optimal treatment strategy to guide clinical decision‐making.

## 2 Methods

According to the Preferred Reporting Items for Systematic Reviews and Meta‐Analyses (PRISMA) guidelines [20], an online search was conducted across three major medical databases: PubMed, Web of Science, and Cochrane. The search strategy is detailed in Table S1. Inclusion criteria consisted of prospective, phase II–III, randomized neoadjuvant clinical trials with at least one arm containing endocrine therapy (ET). Studies with a single treatment arm, HER2+ or triple negative subtypes, inflammatory or metastatic breast cancer, or male breast cancer were excluded. All included articles were published in the English language. This study did not require approval from an academy ethical committee because the analysis involved secondary use of published data and each original study had obtained patients' consent.

The primary endpoint was the clinical response rate (CRR) by physical examination and radiology. Clinical response was defined as the neoplasm achieving complete response (CR) or partial response (PR) according to RECIST criteria [21]. Secondary endpoints were pathological complete response (pCR) and the proportion of patients undergoing breast‐conserving surgery (BCS). Pathological response is defined as no invasive tumor in the breast (ypT0/Tis NX). Additional data, including changes in Ki‐67 at surgery and the preoperative endocrine prognosis index (PEPI), were also collected and analyzed when available [15]. All analyzed data were extracted from intention‐to‐treat (ITT) population source outcomes. Literature review and data extraction were performed individually by two separate authors.

Risk of bias was assessed using the version 2 of the Cochrane tool for assessing risk of bias in randomized trials (RoB 2) in five domains: (1) Selection of the reported result, (2) Measurement of the outcome, (3) Missing outcome data, (4) Deviations from intended interventions, and (5) Randomization process. Each domain was evaluated as having a “low risk of bias”, “some concerns” or “high risk of bias” and an overall risk of bias was determined as low, medium or high [22].

In order to estimate the effect size of binary outcome, odds ratio together with their 95% confidence intervals (CIs) were calculated by Mantel–Haenszel method. Test of global inconsistency was evaluated using design‐by‐treatment‐interaction model and a *p* > 0.05 of Wald test showed no presence of inconsistency [23]. The node‐splitting method evaluates whether the results from direct comparisons and indirect comparisons are consistent with each other, which a *p* > 0.05 in each separate model revealed no local inconsistency occurring in all closed loops. Best treatment was identified using the top ranking of the calculated surface under the cumulative ranking (SUCRA) metric used to measure the effectiveness of each neoadjuvant therapy. The netleague table listed the pooled ORs and 95% CIs among each treatment arm comparison. Publication bias was assessed through the Egger's test and visualized using the funnel plot. All network meta‐analyses were performed using Stata software (version 18).

## 3 Results

**FIGURE 1:**
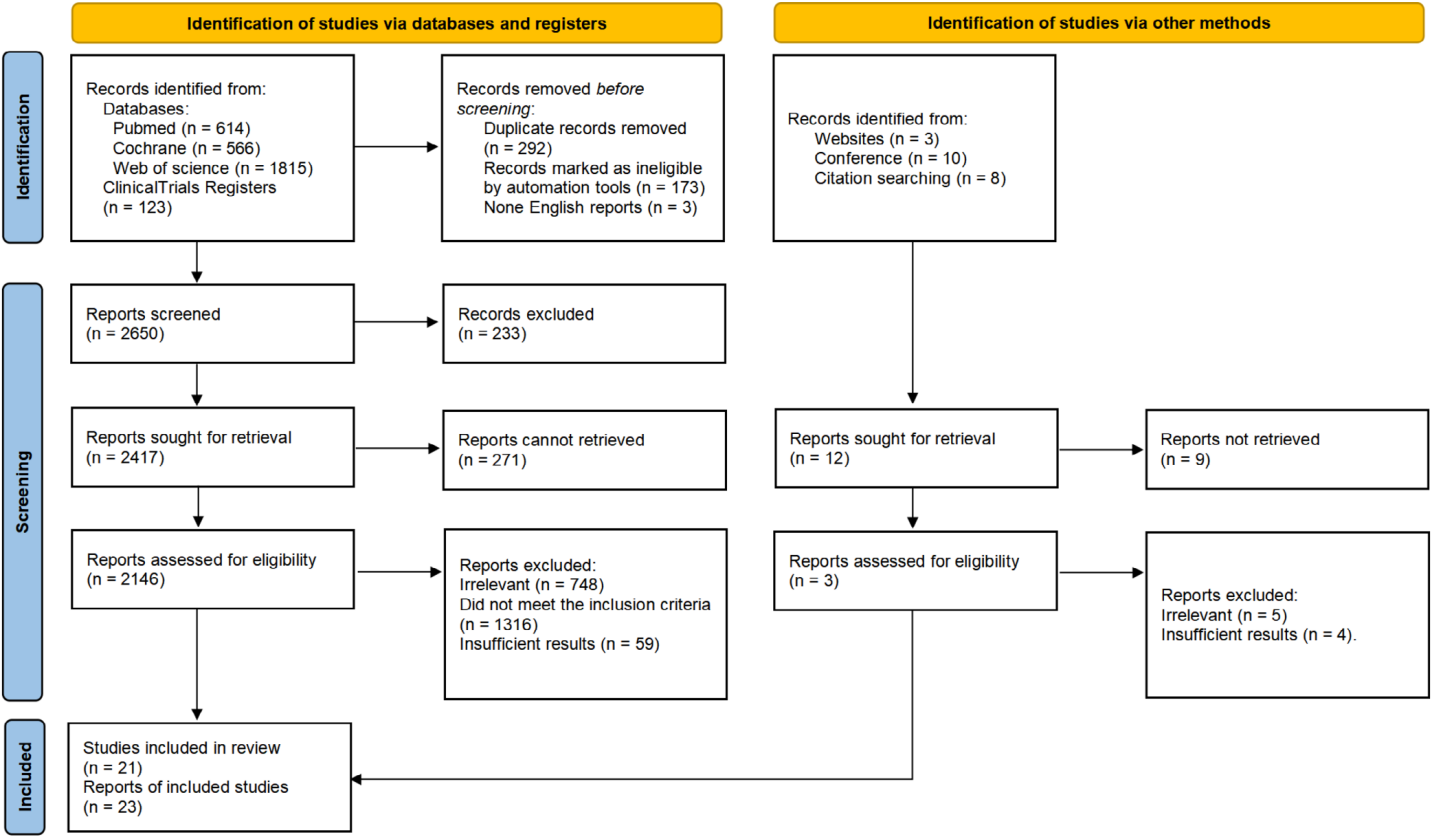
PRISMA Flowchart.

There were in total 21 trials identified for analysis, summing up 5181 patients were included (Figure 1) [24, 25, 26, 27, 28, 29, 30, 31, 32, 33, 34, 35, 36, 37, 38, 39, 40, 41, 42, 43, 44]. Risk of Bias tool presented mild bias, which most of the studies showed a low risk of bias (Table S2). Funnel plot in each analysis also showed no fundamental publication bias (Figure S1). Most of the female patients were post‐menopausal; only 3 trials consisted of both pre‐ and post‐menopausal women, and 2 trials had an inclusion criteria restricted to pre‐menopausal status. Endocrine therapy duration ranged from 12 weeks to 24 weeks, and chemotherapy was given every 3 weeks for 4–8 cycles according to different regimens (A taxane and an anthracycline‐based regimen were given concurrently or sequentially). Pre‐menopausal patients were required to receive ovarian function suppression (OFS) with TAM or AI. Study treatments were manually categorized into 7 groups: Tamoxifen (TAM), Aromatase inhibitors (AIs), Selective Estrogen Receptor Degraders (SERDs), CDK4/6 inhibitors (CDK4/6i) plus ET, tyrosine kinase inhibitors (TKIs) plus ET, and CT. Details of included trials were summarized in Table 1.

**TABLE 1:** Details of included studies.

	Trial	Year	NCT	Center	Menopause	Total participants	Experimental arm	*N*	Duration, weeks	Control arm	*N*	Duration, weeks	Primary endpoint		Secondary endpoint		AE
1	PALLET phase II	February 2015 to March 2018	NCT02296801	38	Post	279	B: Letrozole+Palbociclib from week 2	63	14	A: Letrozole	93	14	clinical response by ultrasound	A versus B versus C versus D versus B + C + D: CR, 2.2% versus 1.6% versus 3.3% versus 1.6% versus 2.2%; PR 47.3% versus 47.6% versus 54.1% versus. 52.2%; SD 45.2% versus 47.6% versus 41.0% versus 38.7% versus 42.5%	pCR	ypT0/Tis ypNx:1.1% versus 1.7% versus 5.0% versus 3.3% versus 3.3%; ypT0/Tis ypN0: 0.0% versus 1.7% versus 1.7% versus 0.0% versus 1.1%	Grade 3+ AEs: A versus B + C + D: 17% versus 50%
							C: Palbociclib + Letrozole from week 2	61	14				Ki67	Median −2.2 versus −4.1 versus −4.0 versus −3.9 versus −4.1	Changes in surgical intent	14.1% versus 14.1%	
							D: Palbociclib + Letrozole	62	14				CCCA at surgery	A versus B + C + D: 58.5% versus 90.4%	BCS	A versus B + C + D:80.4% versus 84.2%	
2	NeoPAL, phase II	February 2015 to November 2016	NCT02400567	22	Post	106	Letrozole + Palbociclib	53	19	Chemotherapy	53	12–18	RCB	RCB 0‐I,7.7% versus 15.7%; RCB II‐III, 92.3% versus 84.3%	ypT0/Tis ypNx	3.8% vs. 5.9%	Grade 3+ AEs: Letrozole + Palbociclib versus Chemotherapy, 39.6% versus 50%; Serious AEs: 3.8% versus 32.1%
															Clinical response	CR 31.4% versus 30%; PR 43.1% versus 46%; SD 25.5% versus 24%	
															BCS	69.2% versus 68.6%	
															PEPI score 0	16% versus 11.5%	
3	CORALLEEN, phase II	July 2017 to December 2018	NCT03248427	21	Post	106	Ribociclib + Letrozole	52	24	Chemotherapy (AC*4‐wPac*4)	54	12	Low‐ROR proportion at surgery	ET versus CT: 46.9% versus 46.1%	clinical response (MRI):	CR 14.3% versus 19.2%; PR 42.9% versus 59.6%; SD 32.6% versus 13.5%	Grade 3+ AEs: 56.9% versus 69.2% Serious AEs: 3.8% versus 32.1%
															pCR	ypT0/Tis ypNx: 2% versus 5.8%; ypT0/Tis ypN0: 0% versus 5.8%	Discontinuation: 15.7% versus 19.2%
															RCB 0‐I:	6.1% versus 11.8%	
															BCS	85.7% versus 72.2%	
															PEPI score 0	17.3% versus 22.5%	
4	FELIN, phase II	February 2016 to August 2018	NCT02712723.	9	Post	120	Ribociclib + Letrozole	82	24	Letrozole	38	24	Rate of PEPI score 0	25% versus 25%			Grade 3+ AEs: 51.2% versus 10%
													CCCA at surgery	71.4% versus 63.3%			
5	SAFIA, phase II	October 2017 to July 2021	NCT03447132	24	Any (RS < 31)	253	Fulvestrant + Palbociclib	106	16	Fulvestrant	111	16	pCR	2% versus 7%, *p* = 0.1464	Radiologic response	Major response (CR + PR) 62% versus 66%; clinical benefit 98% versus 96%	Grade 3–4 neutropenia 25% versus 1.7%
															BCS	47% versus 48%	
6	P024, phase II	March 1998 to August 1999	—	55	Post	324	Letrozole	154	16	Tamoxifen	170	16	Clinical response by palpation	55% versus 36%	Radiologic response at 4 months (mammography)	34% versus 16%	Serious AEs: 0.637% versus 0.588%; Discontinuation: 0.637% versus 0.588%
															Radiologic response at 4 months (ultrasound)	35% versus 25%	
															BCS	45% versus 35%	
7	IMPACT, phase III	October 1997 to October 2002	—	19	Post	330	Tamoxifen + Anastrozole	109	12	Anastrozole	108	12	Clinical response by palpation	39% versus 37% versus 36%	Radiologic response (ultrasound)	28% versus 24% versus 20%	Most common AE‐hot flashes: 28% versus 18% versus 26% Serious AEs: Ana versus Tam 3.7% versus 4.6%
										Tamoxifen	113	12			BCS	24% versus 44% versus 31%	Discontinuation: 1.8% versus 2.8%
8	PROACT, phase III	August 2000 to September 2002	NCT00232661	81	Post	451	Anastrozole±CT	228	12	Tamoxifen ± CT	223	12	Radiologic response (ultrasound)	All patients: OR,39.5% versus 35.4%	Pathologic and caliper responses	All patients: OR, 50% versus 46.2%	Serious AEs: 9.6% versus 7.2%
														Endocrine therapy‐only:36.2% versus 26.5%	BCS	Endocrine therapy‐only:36.2% versus 26.5%	Discontinuation: 0.6% versus 0.7%
																50.3% versus 45.03%	
9	STAGE, phase III	Oct 2007 to May 2009	—	27	Pre	197	Anastrozole + OFS (goserelin)	98	24	Tamoxifen + OFS (goserelin)	99	24	Clinical response by palpation	OR,70.4% versus 50.5%; CR 12.2% versus 7.1%;58.2% versus 43.4%	Histopathological response	Grade 0: 12.2% versus 19.2%; Grade 1a: 42.9% versus 44.4%; Grade 1b: 28.6% versus 18.2%; Grade 1b: 28.6% versus 18.2%; Grade 2: 12.2% versus 9.1%; Grade 3: 1% versus 0%	Treatment‐related adverse events:84% versus 77%; Grade 3+ AEs:2% versus 1%; Serious AEs: 1% versus 0%
													Radiologic response (ultrasound)	OR, 58.2% versus 42.4%; CR 1.0% versus 0%; 57.1% versus 42.4%	BCS	86% versus 68%	
													Radiologic response (MRI or CT):	OR, 64.3% versus 37.4%; CR 2.0% versus 0%;62.2% versus 37.4%			
10	ALTERNATE, phase III	February 2014 to November 2018	NCT01953588	Multicenter	Post	1298	Fulvestrant	430	24	Anastrozole	434	24	pCR	ypT0/Tis ypNX: Anas versus Fulv versus A + F, 1.2% versus 0.9% versus 0.5%; Chemotherapy cohort: pCR 4.8% versus RB‐I 10.2%, RCB 0‐I 15% (25/167)	BCS	Anas versus Fulv versus A + F: 69.6% versus 69.3% versus 70.7%	Grade 3+ AEs: 2.3% versus 0.9%
							A + F	434	24				mPEPI	17.5% versus 21.9% versus 20.0%			
							Patients with a week 4 Ki67 > 10% and switched to NCT	153									
11	NEST, phase III	June 2012 to February 2016	NCT01622361	7	Pre	174	Endocrine therapy (tamoxifen + goserelin)	87	24	Chemotherapy (AC*4‐T*4)	87	24	Clinical response by palpation	OR, 71.3% versus 83.9%; CR,19.5% versus 31%; 51.5% versus 52.9%	pCR	ypT0/Tis ypNx: 1.2% versus 5.7%; ypT0/Tis ypN0: 1.2% versus 3.4%; ypN0: 4.9% versus 13.8%	Grade 3+ AEs: 0% versus 21.8%
													Radiologic response (MRI):	OR, 52.9% versus 83.7%; CR,2.3% versus 16.3%; 50.6% versus 67.4%	BCS	46% versus 55.2%	
12	Semiglazov 2007, phase II	—	—		Post	239	Endocrine therapy (anastrozole/exemestane)	121	12	Chemotherapy (APac*4)	118	12	Clinical response by palpation	OR,64.5% versus 63.6%; CR, 10% versus 10%; PR, 55% versus 53%	Radiologic response (ultrasound)	OR,40% versus 46%; CR, 3% versus 4%; PR, 37% versus 42%	Neutropenia (grade 2–4):0% versus 43%
															Radiologic response (mamography)		
															pCR	3% versus 6%	
															BCS	46% versus 55.2%	
13	Semiglazov 2005, phase II	—	—	\	Post	151	Exemestane	76	12	Tamoxifen	75	12	Clinical response	OR, 76.3% versus 40%	Radiologic response (ultrasound)	64% versus 37.3%	
															Radiologic response (mamography)	60.5% versus 37.3%	
															BCS	36.8% versus 20%	
															3 years‐DFS	78.9% versus 74.6%	
14	LORELEI, phase II	November 2014 to August 2016	NCT02273973	85	Post	334	Taselisib + Letrozole	166	16	Letrozole	168	16	Radiologic response (MRI)	OR, 50% versus 39%; CR, 5% versus 2%; 45% versus 38%; SD,40% versus 51%	Radiological response (MRI) in PIK3CA wild‐type patients/PIK3CA mutant patients	Wild‐type: OR,46% versus 40%; CR, 3% versus 1%; PR,42% versus 39%; SD,41% versus 53%; mutant: OR, 56% versus 38%; CR, 7% versus 3%; PR, 49% versus 38%; SD, 38% versus 49%	Grade 3+ AEs: 26% versus 8%; Serious AEs: 12% versus 2%
													pCR	ypT0/Tis ypN0: 2% versus 1%	pCR in PIK3CA wild‐type patients	Wild‐type: 2% versus 1%; mutant,1% versus 0%	Discontinuation: 10.8% versus 2.4%
															change in Ki67 expression	baseline to week 3:‐83.81% versus −80.44%; baseline to surgery: −75.58% to −80.51%	
15	NEOLBC phase II	June 2019 to March 2026 (estimate)	NCT03283384	29	Post	66	Letrozole + Ribociclib	34	24	Chemotherapy (AC*4‐wPac*12)	32	24	CCCA (Ki67 < 1%) at surgery	70% versus 35%	pCR	ypT0/Tis ypNx: 11.8% versus 3.1%; ypT0/Tis ypN0: 8.8% versus 3.1%	Serious AEs: 6.3% versus 3.1%; Discontinuation due to toxicity:23.5% versus 31.3%
16	CARABELA, phase II	October 2020 to June 2023	NCT04293393	24	Any	200	Letrozole + OFS + abemaciclib	100	48	Chemotherapy (Anthracyclines plus taxanes regimens)	100	24	RCB 0‐I	13% versus 18%	pCR	ypT0/Tis ypN0: 4% versus 10%	Discontinuation by AEs: 9% versus 4%
													RCB	RCB‐I, 9% VS 8%; RCB‐II, 57% VS 56%; RCB‐III: 22% VS 23%; *p* = 0.70	CRR	78% versus 71%, *p* = 0.26	
															PEPI score 0	14% versus 26%, *p* = 0.03	
17	GEICAM/2006–03, phase II	March 2007 to December 2008	NCT00432172	15	Any	95	Exemestane	48	24	Chemotherapy (EC*4‐T*4)	47	16	Clinical response by palpation	OR, 48% versus 66%,*p* = 0.075; CR,13.0% versus 6.0%; PR, 53% versus 42%; SD, 28% versus 40%	pCR	ypT0/Tis ypN0: 2.1% versus 0%	Grade 3+ AEs: 9% versus 47%; Serious AEs: 0% versus 15.6%
														OR: premenopausal 44% versus 75%; postmenopausal 52% versus 57%	BCS	56.3% versus 46.8%	Discontinuation: 8.9% versus 10.9%
18	IL1839/223, phase II	—	—		Post	206	Anastrozole + Gefitinib	31 90	16 16	Anastrozole	85	16	Change in Ki67 expression	Baseline to 16 weeks: A + B versus C, −77.4% versus −83.6%, *p* = 0.26; baseline to 2 weeks: A versus B + C, −80.1% versus −71.3%, *p* = 0.22; week 2 to 6: B versus C: −19.3% versus −43.3%, *p* = 0.16	Clinical response	A + B versus C: OR, 48% versus 61%; CR, 7% versus 4%; PR, 40% versus 57%, SD, 37% versus 33%.	Discontinuation by AEs: 13% versus 2%
							Anastrozole 2 weeks → Anastrozole + Gefitinib 14 weeks								BCS	A + B versus C: 61.8% versus 44.6%	
19	Guarneri 2014, phase IIB	August 2006 to June 2012	NCT00429299		Post	92	Letrozole + Lapatinib	43	24	Letrozole	49	24	Radiologic response (any)	OR, 70% versus 63%； CR, 12% versus 2%；PR, 58% versus 61%; SD 23% versus 29%	pCR	None	Grade 3+ AEs: 44.1% versus 2%
													Radiologic response (ultrasound)	OR, 69.2% versus 57.8%	BCS	61.8% versus 44.6%	Discontinuation: 11.6% versus 8.2%
20	NEOCENT, phase III	November 2008 to March 2011	NCT00963729	13	Post	44	Letrozole	22	18–23	Chemotherapy (FEC*6) or switch to T*3 after 3 cycles	22	18	Radiologic response (ultrasound/mammogram)	OR, 59.1% versus 54.5%; CR 0% versus 9.1%; PR, 59.1% versus 45.5%	pCR	None	Grade 3+ AEs: 0% versus 50%; Serious AEs: 0% versus 40%
													clinical response by palpation	OR, 90.9% versus 77.3%; CR, 0% versus 13.6%; PR, 90.9% versus 63.6%	BCS	68.2% versus 54.6%	Discontinuation: 9.1% versus 9.1%
21	CARMINA 02, phase II	October 2007 to April 2011	NCT00629616	Multicenter	Post	116	Anastrozole	59	16–24	Fulvestrant	57	16–24	Clinical response by palpation	OR, 52.6% versus 36.8%	pCR	17.8% versus 14.3%, *p* = 0.79	Grade 3+ AEs: 1.7% versus 0%; Serious AEs: none
															Radiologic response (ultrasound)	OR, 54.4% versus 54.1%	Discontinuation: none
															Radiologic response (MRI)	OR, 53.5% versus 58.5%	
															BCS	68.2% versus 54.6%	
															PEPI score 0	10.5% versus 19.0%	
															3y‐RFS	94.9% versus 91.2%	
															3y‐OS	100% versus 98%	

### 3.1 Overall Response Rate

**FIGURE 2:**
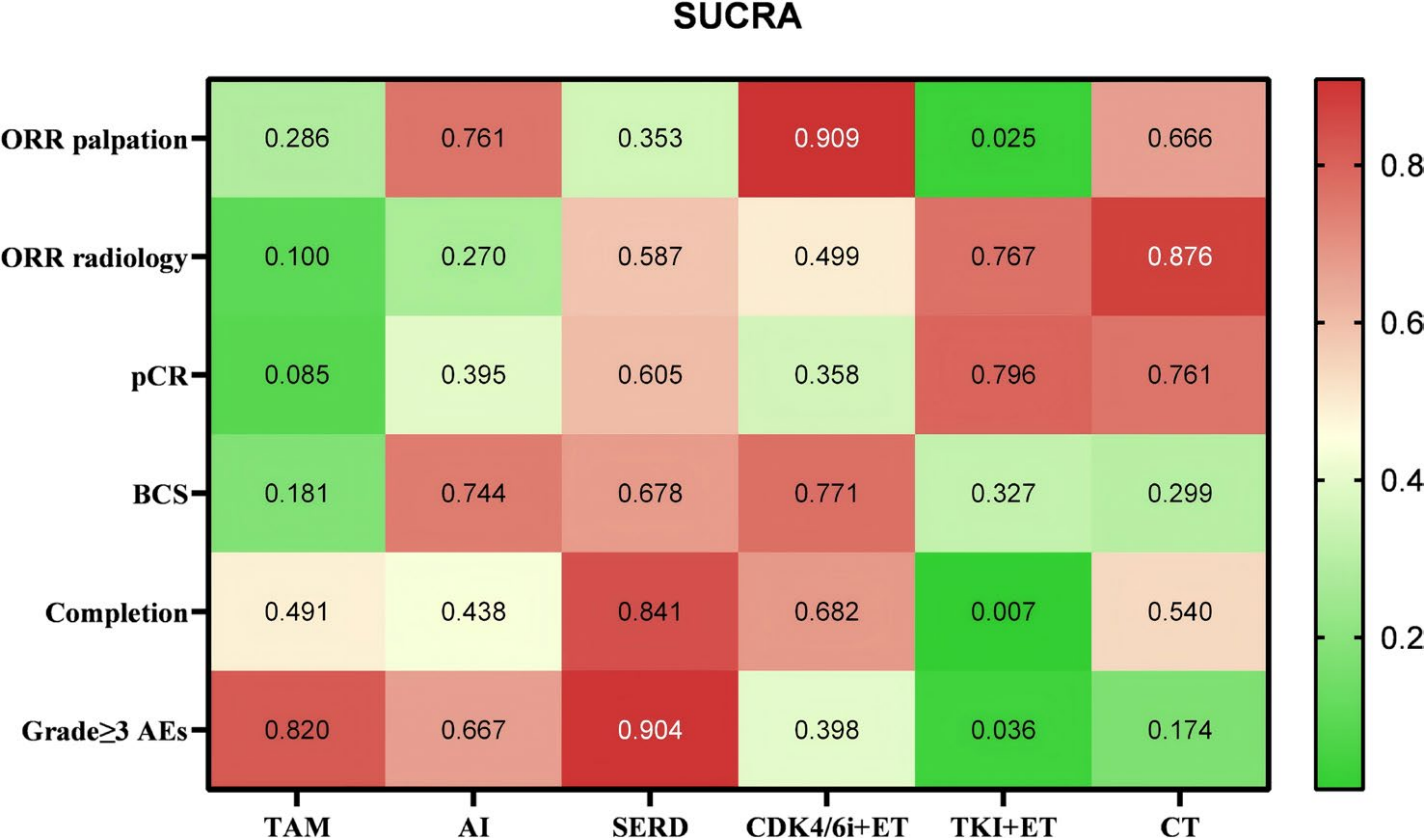
SUCRA score for multiple study endpoints.

19 trials were qualified for the analysis of the primary endpoint, network plot revealed the comparison between each intervention among included trials (Figure S2A,B). Figure 2 presented the overall SUCRA rankings for each study endpoint, where a higher score corresponds to a more favorable outcome (Gradient colors ranging from deep red to light and to deep green correspond to a progressive decrease in numerical score values). Tumor evaluation by palpation was obtained in 14 studies and network analysis showed that tumors had better clinical response to CDK4/6i plus ET than others (ORs range 1.17–2.44, Table S3). Unfortunately, none of the differences were statistically significant. Additionally, radiographic evaluation by ultrasound or MRI in 16 studies showed that CT presented significantly higher incidences of radiographic response than AI and TAM (CT vs. AI, OR = 1.65, 95% CI = 1.20–2.27; CT vs. TAM, OR = 3.04, 95% CI = 2.08–4.44). The same trend could also be found when compared CT to other types of treatments but the results were insignificant (ORs range 1.10–1.37, Table S4).

Score of SUCRA indicated that CDK4/6i + ET had the highest probability of being the best treatment arm for clinical response by palpation (SUCRA = 0.824), followed by AI alone (SUCRA = 0.747), and CT (SUCRA = 0.595), while TAM had the lowest probability (SUCRA = 0.173) (Figure 2). The SUCRA score of radiographic response showed distinct ranking, which CT had the highest probability of being the best treatment arm (SUCRA = 0.872), followed by TKI + ET (SUCRA = 0.783), and SERD (SUCRA = 0.583), while TAM consistently showed the lowest probability (SUCRA = 0) (Figure 2).

**FIGURE 3:**
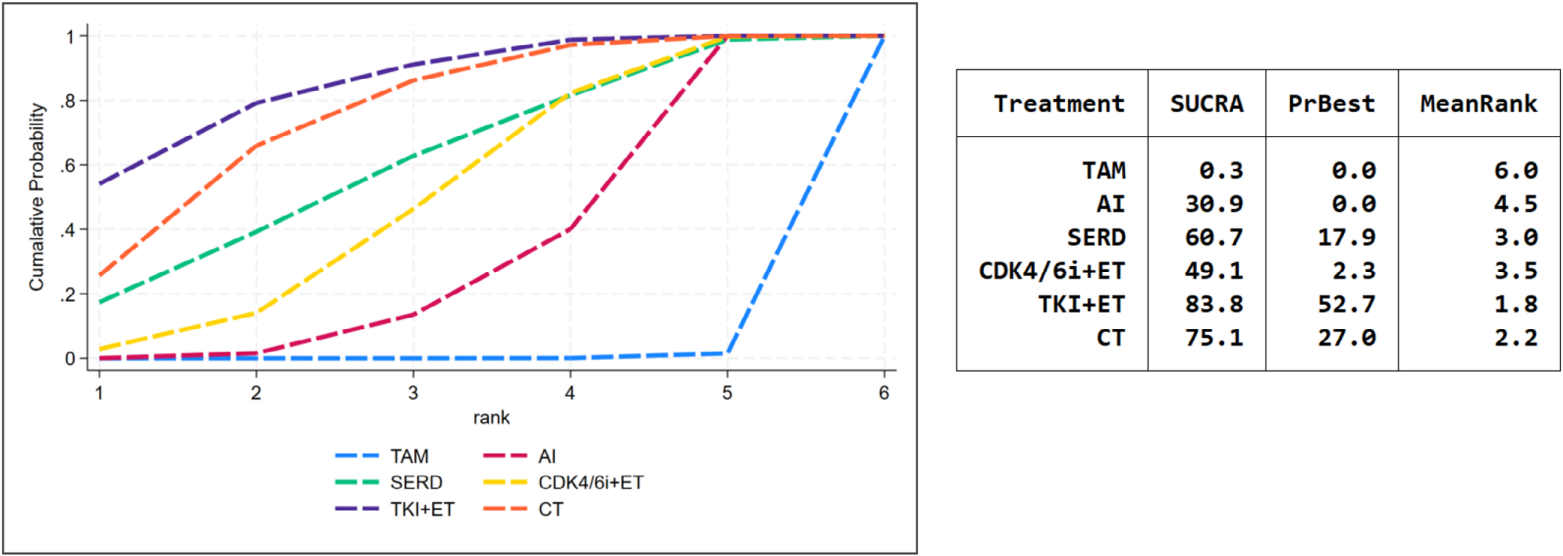
SUCRA score for overall response by radiography in postmenopausal subgroup.

Subgroup analysis looked into post‐menopausal women, the SUCRA score of radiographic response still saw TKI + ET as the best treatment (SUCRA = 0.846, Figure 3). The comprehensive ORs also showed a tendency toward superior response, but the comparisons were not statistically significant compared TKI + ET to SERD, CDK4/6 + ET, and CT (ORs range 1.11–2.47, Table S5).

### 3.2 Pathological Complete Response

Only 14 trials reported the pCR outcome where the network plot revealed that most studies were centered around AI treatment (Figure S2C). The results were similar to previous literature demonstrating a low pCR rate for HR+/HER2− BC, which ranged from 0% to 11.8% among all types of therapy. The highest pCR ratio was seen in the CDK4/6i + ET (Ribociclib plus letrozole) cohort of the NEOLBC trial. Paired comparisons showed no significance, but there were uniform trends pointing to a better outcome when comparing TKI + ET to others (ORs range 1.76–6.73, Table S6).

Same conclusions could also be seen in the SUCRA score where TKI calculated the top ranking that indicated the highest probability for best pathological response (SUCRA = 0.796). The score of CT was closely similar (SUCRA = 0.761), which ranked the second, next followed by SERD (SUCRA = 0.605) (Figure 2).

### 3.3 Breast Conservation Surgery

The ratio of patients accepting breast conservation surgery indicates an observational outcome synthesized from both clinical and pathological metrics. Details of types of surgery were extracted from 16 trials (Figure S2D). Resemble to clinical response, patients assigning to CDK4/6i + ET treatment had higher chance to succeed in BCS but the superiority was not statistically significant (ORs range 1.04–1.06, Table S7).

SUCRA ranking revealed that CDK4/6i + ET was the most efficient treatment that allows for mastectomy remission (SUCRA = 0.771), followed by AI (SUCRA = 0.744), and SERD (SUCRA = 0.678) (Figure 2).

### 3.4 Completion

The treatment completion rate would affect the study outcome. Most patients had completed a full cycle of therapy, and the average discontinuation rate was 8.8% among all study groups. Only the CT and CDK4/6i + ET groups from 2 studies had reported more than a 15% proportion of patients requesting intermediate drop‐out due to intolerability, and the CT group in the NEOLBC trial reported the most patients discontinued (10/32, 31.3%) (Figure S2E). Figure 2 showed that SERD had the highest probability for treatment adherence (SUCRA = 0.825) and TKI scored the lowest (SUCRA = 0.071).

Grade ≥ 3 drug‐related adverse events (AEs) were the main reason for treatment discontinuation. The incidence rate varied widely across studies and treatment arms, ranging from 0% to 70% (Figure S2F). Single drug ET (TAM, AI, and SERD) accounted for a relatively lower severe AEs incidence, and SERD was estimated to be the most safety treatment (SUCRA = 0.904, Figure 2). Paired‐wise comparison ORs were listed in Tables S8 and S9.

### 3.5 Cell Cycle Arrest and PEPI

**FIGURE 4:**
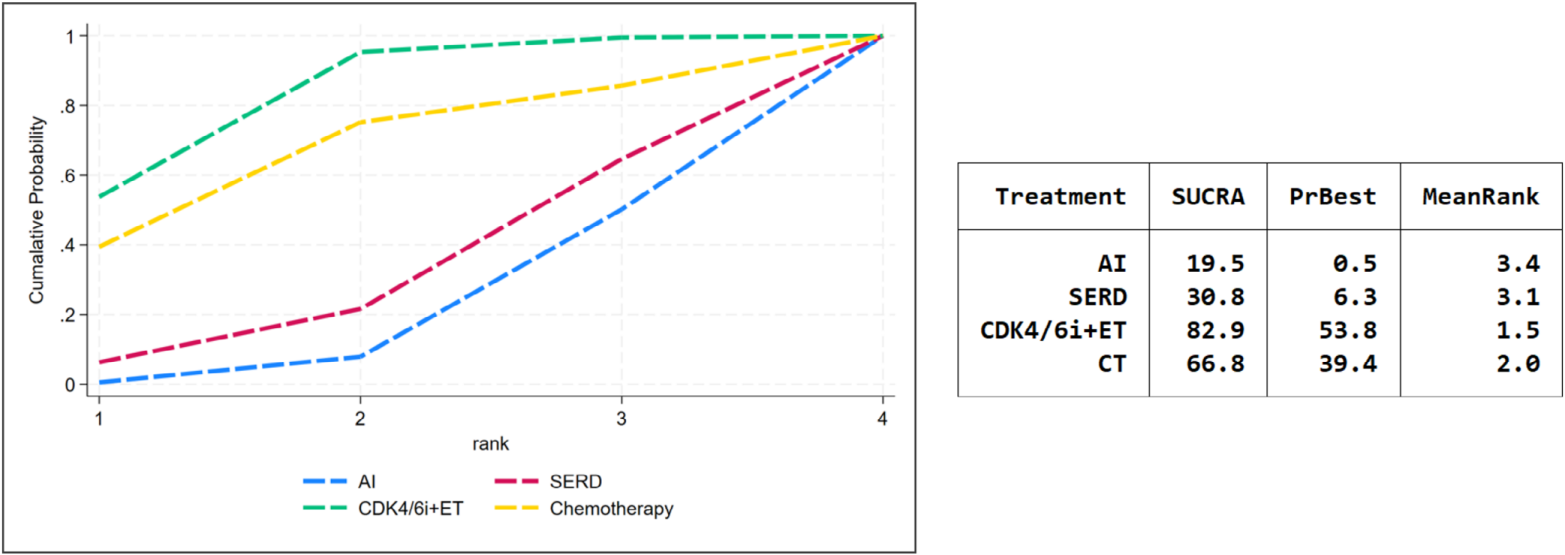
SUCRA score for complete cell cycle arrest.

**FIGURE 5:**
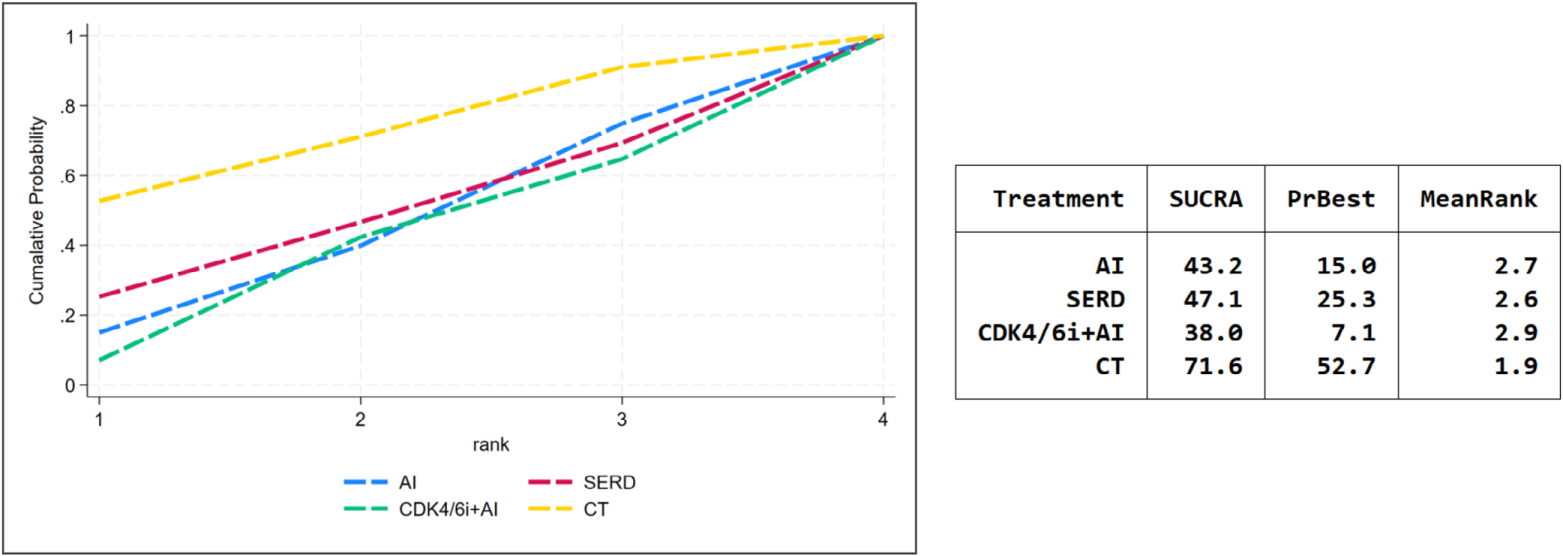
SUCRA score for PEPI score 0.

Exploratory analysis looked into alternative outcomes of NET: the suppression of Ki‐67 and the PEPI score. Complete cell cycle arrest (CCCA) was defined as Ki‐67 ≤ 2.7%. 5 studies had examined the rates of CCCA and CDK4/6i + ET had outperformed the best in Ki‐67 suppression (SUCRA = 82.9, Figure 4). Furthermore, we observed that the proportion of patients achieved PEPI = 0 was most obvious in CT group (SUCRA = 71.6), followed by SERD (SUCRA = 47.1) and AI (SUCRA = 43.2). CDK4/6i + ET had failed to received better performance in PEPI among four treatments and rank the lowest SUCRA score (SUCRA = 38.0) (Figure 5).

## 4 Discussion

Our study did not identify a single significantly superior treatment which Figure 2 showed a scattered distribution of red squares (representing high SUCRA scores). However, NCT demonstrated strong tumor inhibition, supported by its relatively outstanding SUCRA score across most study outcomes. Unlike HER2+ and triple negative breast cancer (TNBC), the HR+/HER2− subtype typically exhibits indolent growth and a favorable prognosis [45]. While pCR is a common endpoint in neoadjuvant studies, it remains consistently low in HR+/HER2− patients and has limited correlation with long‐term prognosis [13, 46]. Thereby, we primarily evaluated therapeutic efficacy using presurgical tumor reduction metrics (ORR and BCS rate).

Notably, clinical response and BCS rate yielded concordant results in identifying the best treatment. These parameters reflect the likelihood of achieving optimal cosmetic outcomes while minimizing therapeutic intervention. Our results demonstrated that CDK4/6i + ET had the highest probability of achieving the best clinical ORR and BCS rates. Additionally, among all dual therapies and CT, CDK4/6i‐based treatment was the best tolerated, with the lowest SUCRA score for grade 3+ AEs. However, radiographic response did not fully align with clinical response (assessed by palpation) where CT and TKI + ET emerged as the most effective treatments—consistent with the pCR ranking. Bypass signaling pathways, such as epidermal growth factor receptor (EGFR) and HER‐2 abnormal activity, can contribute to endocrine resistance, whereas combination therapies may completely block dual‐pathway crosstalk. Nonetheless, the lower treatment completion rate and more severe adverse incidents make it hard to balance the risks and benefits.

For luminal breast cancer, neoadjuvant therapy provides a window to assess endocrine sensitivity. The neoMONARCH trial randomized patients to a 2‐week lead‐in therapy and found that patients resistant to anastrozole could restore endocrine sensitivity by adding abemaciclib [47]. Cyclin‐dependence kinase 4 and 6 are key regulatory proteins that drive cell cycle progression from the G1 phase to the S phase [48]. Specific inhibitors target the catalytic activity of these kinases, suppressing RB phosphorylation of RB and preventing the release of E2F transcription factors required for initiating S‐phase entry [49]. CDK4/6 inhibitors block the cyclinD‐CDK4/6 complex, thereby inhibiting ER‐dependent abnormal activation and cell cycle progression [2, 50]. Our exploratory analysis found that the CDK4/6i + ET group had the highest likelihood of achieving CCCA, consistent with the results of neoMONARCH trial demonstrating that short‐term CDK4/6i combination therapy induces potent cell‐cycle inhibition. Although we did not observe the same benefits of CDK4/6i in the PEPI score, differences between each treatment groups were subtle. The PEPI score is a composite index incorporating pathologic tumor size, lymph node status, Ki67 level, and ER Allred score. However, the inclusion of the ER Allred score in PEPI remains controversial. Patients may lose ER expression following endocrine therapy, and this could explain the relatively poor PEPI performance observed in the AI and CDK4/6i + AI groups [51]. The modified PEPI (mPEPI), which excludes the ER Allred score, may better predict response to neoadjuvant endocrine therapy, though only one study has reported on this to date [33]. Selection bias is also a potential concern, as patients receiving CDK4/6i + AI may have presented with worse baseline tumor characteristics (e.g., larger tumor size and greater lymph node involvement) compared to other groups.

High risk HR+/HER2− patients may exhibit biological features similar to those with advanced disease, inherently conferring resistance to TAM or AI [52]. SERDs are universally used in metastatic luminal breast cancer or after resistance to first‐line endocrine therapy [53, 54]. In our study, however, SERDs demonstrated clear benefits, showing favorable performance across all endpoints along with high tolerability. The optimal sequencing of SERDs remains uncertain, as insufficient evidence supports prioritizing SERDs over AIs or TAM in early‐stage disease. Additionally, SERD‐induced ER degradation alters tumor biology by reducing ER expression [55], yet no consensus exists on whether SERDs should be continued as postoperative maintenance therapy or on their proper treatment duration. Fulvestrant, administered via intramuscular injection, poses challenges for long‐term use due to potential adherence issues. Oral SERDs like Elacestrant offer a more convenient alternative, though current evidence is limited to late‐line advanced breast cancer [56].

For high‐risk luminal breast cancer, neoadjuvant therapy is primarily used to assess treatment sensitivity or achieve tumor downstaging for breast‐conserving surgery in most patients. The collection of tumor samples at separate timepoints before and after treatment enables comprehensive analysis of predictive biomarkers. In 2023, the St.Gallen consensus experts acknowledged that short‐term (2–4 weeks) NET‐induced reduction in Ki‐67 levels could serve as valuable guidance for omitting postoperative chemotherapy [57]. The ADAPT trial demonstrated that a 21‐gene recurrence score RS ≤ 11 or RS12‐25 combined with ET response (Ki67_post_ ≤ 10%) could thus spare CT in patients with fewer than 3 involved lymph nodes [58].

This study has several limitations. First, the included trials predominantly enrolled postmenopausal patients. While we found high consistency between the overall population and postmenopausal subgroup, the limited number of premenopausal patients prevents extrapolation of conclusions to both menopausal statuses. Premenopausal breast cancer exhibits unique characteristics, with more aggressive biological features. Consequently, it often carries a higher risk of recurrence, poorer prognosis, and increased mortality. Therefore, greater attention should be paid to the appropriateness of drug selection. Second, endocrine therapy durations varied across trials. Most trials controlled treatment duration in both arms, typically matching chemotherapy duration (range: 14–24 weeks; average 18 weeks). Available evidence suggested that extending treatment duration may improve clinical response but shows no significant benefit for pathological response, with only marginal differences observed between 6‐month to 4‐month duration [17, 44]. The long‐term effects of endocrine therapy create challenges in selecting the optimal treatment window. Whether extending the duration of preoperative endocrine therapy leads to better tumor shrinkage remains unknown. Therefore, future research is still needed to address this question. Third, while inclusion criteria varied between trials, none of the low‐risk patients were enrolled. We made every effort to balance these differences, though this necessitated excluding some relevant prospective clinical trials.

## 5 Conclusions

In our study, CDK4/6i + ET emerged as an optimal option for neoadjuvant therapy based on SUCRA scores, while maintaining tolerability. Mono therapy AI or SERD were encouraged if CDK4/6i intolerance. To further optimize treatment strategies, potential biomarkers should be incorporated to guide decisions regarding chemotherapy use and the duration of postoperative oral endocrine therapy.

## Author Contributions

**Xinyu Li:** conceptualization (equal), data curation (equal), formal analysis (equal), investigation (equal), methodology (equal), project administration (equal), resources (equal), software (equal), visualization (equal), writing – original draft (equal). **Peijing Du:** formal analysis (equal), formal analysis (equal), investigation (equal), investigation (equal), project administration (equal), project administration (equal), software (equal), software (equal). **Tao Huang:** data curation (equal), project administration (equal), supervision (lead), validation (lead), writing – review and editing (lead).

## Funding

The authors have nothing to report.

## Ethics Statement

The authors have nothing to report.

## Consent

The authors have nothing to report.

## Conflicts of Interest

The authors declare no conflicts of interest.

## Supporting information


**Figure S1:** Funnel plot showing risk of publication bias among study endpoints.

**Figure S2:** Network plot of included studies.

**Table S1:** Search strategies of included studies.

**Table S2:** Risk of bias.

**Table S3:** League table showing comparative efficacy of overall response by palpation.

**Table S4:** League table showing comparative efficacy of overall response by radiography.

**Table S5:** League table showing comparative efficacy of overall response by radiography in postmenopausal subgroup.

**Table S6:** League table showing comparative efficacy of pCR.

**Table S7:** League table showing comparative efficacy of breast conservation therapy.

**Table S8:** League table showing comparative efficacy of completion rate.

**Table S9:** League table showing comparative efficacy of less adverse events (≥ grade 3).

## Data Availability

The authors have nothing to report.
