# Supplementary figures and images for: Beyond Chemotherapy: Network Meta‐Analysis Reveals Optimal Neoadjuvant Strategies for Luminal Breast Cancer
Source: Cancer Med. 2026 Feb 13;15(2):e71648. doi: 10.1002/cam4.71648 (PMC12902795; doi:10.1002/cam4.71648)

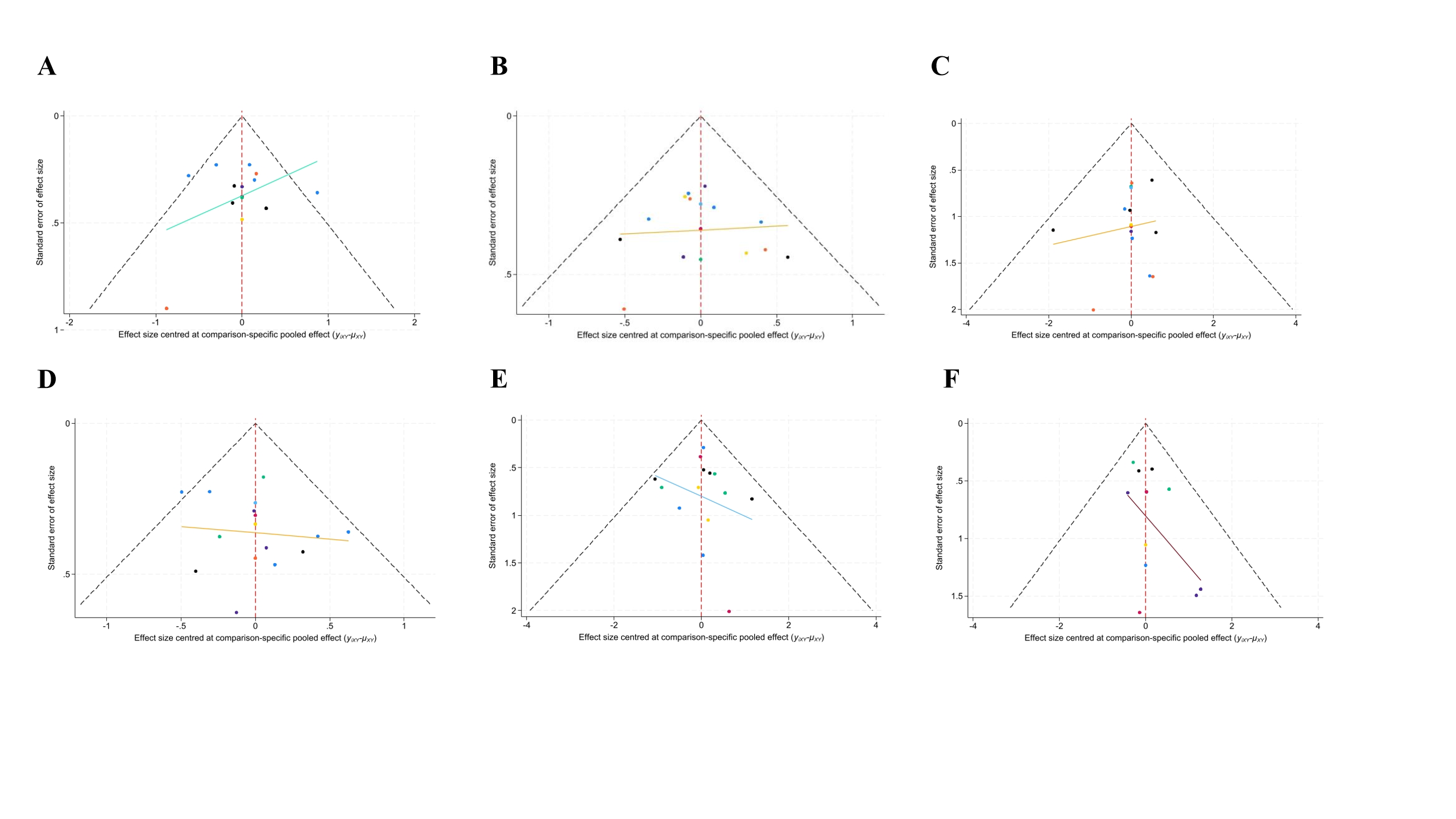

Supplement: Supplementary file 1 — Figure S1: Funnel plot showing risk of publication bias among study endpoints. [file CAM4-15-e71648-s004.tif]

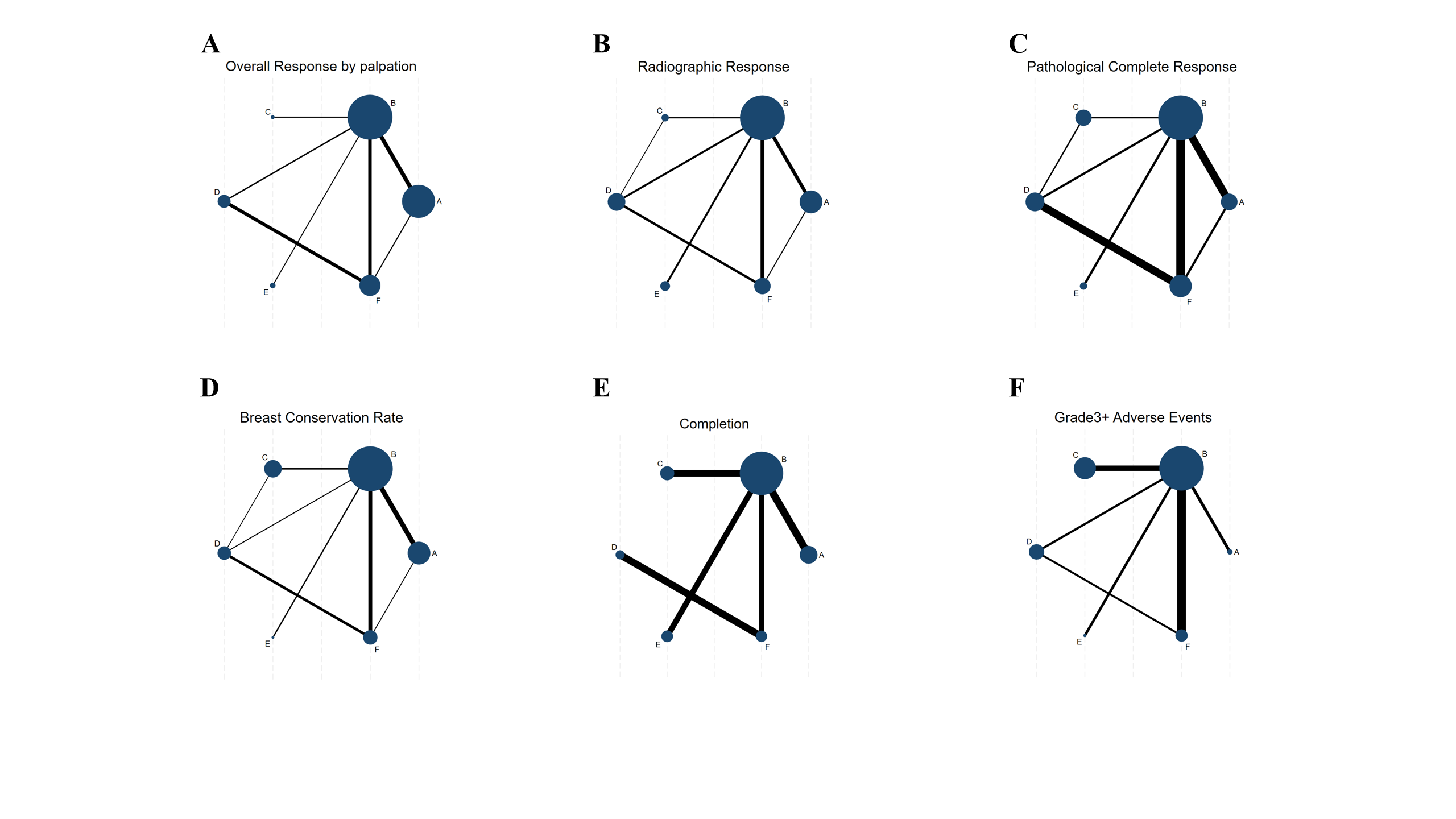

Supplement: Supplementary file 2 — Figure S2: Network plot of included studies. [file CAM4-15-e71648-s009.tif]
