# Supplementary material for: Beyond Chemotherapy: Network Meta‐Analysis Reveals Optimal Neoadjuvant Strategies for Luminal Breast Cancer
Source: Cancer Med. 2026 Feb 13;15(2):e71648. doi: 10.1002/cam4.71648 (PMC12902795; doi:10.1002/cam4.71648)
Supplement: Supplementary file 3 — Table S1: Search strategies of included studies. [file CAM4-15-e71648-s003.docx]

| Search strategies |
| --- |
| (Breast [tw] or Mammary Gland[tw]) AND (Tumor[tw] or Neoplasm[tw] or Mass[tw] or Malignancy[tw] or Cancer[tw]) AND (Luminal[MeSH] or Hormone Receptor-Positive[tw] or Estrogen Receptor-Positive) AND (HER2-negative[tw] or Human Epidermal Receptor-Negative[tw] or ERBB2[MeSH]) AND (Preoperative[tw] or Neoadjuvant[tw] or Neoadjuvant chemotherapy[tw] or Neoadjuvant endocrine therapy[tw]) |
