# Supplementary material for: Beyond Chemotherapy: Network Meta‐Analysis Reveals Optimal Neoadjuvant Strategies for Luminal Breast Cancer
Source: Cancer Med. 2026 Feb 13;15(2):e71648. doi: 10.1002/cam4.71648 (PMC12902795; doi:10.1002/cam4.71648)
Supplement: Supplementary file 5 — Table S3: League table showing comparative efficacy of overall response by palpation. [file CAM4-15-e71648-s010.docx]

Supplementary Table 3. League table showing comparative efficacy of overall response by palpation

| CDK4/6 inhibitors + ET | 0.82 (0.44,1.53) | 0.75 (0.46,1.22) | 0.43 (0.14,1.30) | 0.41 (0.21,0.79) | 0.18 (0.06,0.52) |
| --- | --- | --- | --- | --- | --- |
| 1.22 (0.65,2.29) | AIs | 0.92 (0.55,1.53) | 0.52 (0.21,1.30) | 0.50 (0.36,0.69) | 0.22 (0.10,0.51) |
| 1.33 (0.82,2.16) | 1.09 (0.65,1.81) | TKI + ET | 0.57 (0.20,1.62) | 0.54 (0.31,0.93) | 0.24 (0.09,0.65) |
| 2.33 (0.77,7.03) | 1.90 (0.77,4.73) | 1.75 (0.62,4.96) | SERDs | 0.94 (0.36,2.48) | 0.43 (0.12,1.46) |
| 2.47 (1.27,4.80) | 2.02 (1.46,2.79) | 1.85 (1.08,3.19) | 1.06 (0.40,2.78) | Tamoxifen | 0.45 (0.19,1.10) |
| 5.46 (1.93,15.46) | 4.47 (1.95,10.24) | 4.10 (1.55,10.86) | 2.35 (0.69,8.03) | 2.22 (0.91,5.40) | Chemotherapy |

*ET, endocrine therapy; AIs, aromatase inhibitors; TKIs, tyrosine kinase inhibitors; SERDs, selective estrogen receptor degraders; CT, chemotherapy.
