# Supplementary material for: Beyond Chemotherapy: Network Meta‐Analysis Reveals Optimal Neoadjuvant Strategies for Luminal Breast Cancer
Source: Cancer Med. 2026 Feb 13;15(2):e71648. doi: 10.1002/cam4.71648 (PMC12902795; doi:10.1002/cam4.71648)
Supplement: Supplementary file 6 — Table S4: League table showing comparative efficacy of overall response by radiography. [file CAM4-15-e71648-s011.docx]

Supplementary Table 4. League table showing comparative efficacy of overall response by radiography

| Chemotherapy | 0.91 (0.55,1.51) | 0.78 (0.44,1.38) | 0.73 (0.49,1.08) | 0.61 (0.44,0.84) | 0.33 (0.23,0.48) |
| --- | --- | --- | --- | --- | --- |
| 1.10 (0.66,1.81) | TKIs + ET | 0.85 (0.44,1.64) | 0.80 (0.48,1.34) | 0.67 (0.45,0.98) | 0.36 (0.22,0.58) |
| 1.29 (0.72,2.29) | 1.18 (0.61,2.26) | SERDs | 0.94 (0.59,1.51) | 0.78 (0.46,1.32) | 0.42 (0.24,0.76) |
| 1.37 (0.92,2.03) | 1.25 (0.74,2.10) | 1.06 (0.66,1.71) | CDK4/6 inhibitors + ET | 0.83 (0.59,1.17) | 0.45 (0.29,0.69) |
| 1.65 (1.20,2.27) | 1.50 (1.02,2.22) | 1.28 (0.76,2.16) | 1.20 (0.85,1.69) | AIs | 0.54 (0.41,0.71) |
| 3.04 (2.08,4.44) | 2.77 (1.73,4.45) | 2.36 (1.31,4.24) | 2.22 (1.45,3.39) | 1.85 (1.41,2.41) | Tamoxifen |

*ET, endocrine therapy; AIs, aromatase inhibitors; TKIs, tyrosine kinase inhibitors; SERDs, selective estrogen receptor degraders; CT, chemotherapy.
