# Supplementary material for: Beyond Chemotherapy: Network Meta‐Analysis Reveals Optimal Neoadjuvant Strategies for Luminal Breast Cancer
Source: Cancer Med. 2026 Feb 13;15(2):e71648. doi: 10.1002/cam4.71648 (PMC12902795; doi:10.1002/cam4.71648)
Supplement: Supplementary file 7 — Table S5: League table showing comparative efficacy of overall response by radiography in postmenopausal subgroup. [file CAM4-15-e71648-s001.docx]

Supplementary Table 5. League table showing comparative efficacy of overall response by radiography in postmenopausal subgroup.

| TKIs + ET | 0.90 (0.53,1.55) | 0.81 (0.42,1.57) | 0.75 (0.45,1.27) | 0.67 (0.45,0.98) | 0.40 (0.25,0.65) |
| --- | --- | --- | --- | --- | --- |
| 1.11 (0.65,1.89) | Chemotherapy | 0.90 (0.50,1.63) | 0.83 (0.55,1.26) | 0.74 (0.51,1.07) | 0.45 (0.28,0.71) |
| 1.23 (0.64,2.37) | 1.11 (0.61,2.01) | SERDs | 0.92 (0.58,1.48) | 0.82 (0.48,1.39) | 0.50 (0.27,0.90) |
| 1.33 (0.79,2.24) | 1.20 (0.80,1.82) | 1.08 (0.67,1.74) | CDK4/6 inhibitors + ET | 0.89 (0.62,1.25) | 0.54 (0.35,0.84) |
| 1.50 (1.02,2.22) | 1.36 (0.94,1.97) | 1.22 (0.72,2.07) | 1.13 (0.80,1.60) | AIs | 0.61 (0.46,0.80) |
| 2.47 (1.54,3.97) | 2.23 (1.41,3.54) | 2.01 (1.11,3.64) | 1.86 (1.19,2.89) | 1.64 (1.25,2.16) | Tamoxifn |

*ET, endocrine therapy; AIs, aromatase inhibitors; TKIs, tyrosine kinase inhibitors; SERDs, selective estrogen receptor degraders; CT, chemotherapy.
