# Supplementary material for: Beyond Chemotherapy: Network Meta‐Analysis Reveals Optimal Neoadjuvant Strategies for Luminal Breast Cancer
Source: Cancer Med. 2026 Feb 13;15(2):e71648. doi: 10.1002/cam4.71648 (PMC12902795; doi:10.1002/cam4.71648)
Supplement: Supplementary file 8 — Table S6: League table showing comparative efficacy of pCR. [file CAM4-15-e71648-s005.docx]

Supplementary Table 6. League table showing comparative efficacy of pCR

| TKI + ET | 0.57 (0.05,6.49) | 0.45 (0.04,5.55) | 0.33 (0.03,3.16) | 0.31 (0.03,3.66) | 0.15 (0.01,1.90) |
| --- | --- | --- | --- | --- | --- |
| 1.76 (0.15,20.08) | Chemotherapy | 0.79 (0.25,2.52) | 0.57 (0.24,1.37) | 0.54 (0.25,1.17) | 0.26 (0.07,0.95) |
| 2.21 (0.18,27.19) | 1.26 (0.40,3.99) | SERDs | 0.72 (0.25,2.08) | 0.69 (0.24,1.99) | 0.33 (0.07,1.50) |
| 3.07 (0.32,29.85) | 1.75 (0.73,4.17) | 1.39 (0.48,4.00) | AIs | 0.95 (0.36,2.51) | 0.46 (0.14,1.44) |
| 3.23 (0.27,38.28) | 1.84 (0.86,3.94) | 1.46 (0.50,4.25) | 1.05 (0.40,2.77) | CDK4/6 inhibitors + ET | 0.48 (0.12,1.95) |
| 6.73 (0.53,86.07) | 3.83 (1.05,13.95) | 3.04 (0.67,13.85) | 2.19 (0.69,6.92) | 2.08 (0.51,8.46) | Tamoxifen |

*ET, endocrine therapy; AIs, aromatase inhibitors; TKIs, tyrosine kinase inhibitors; SERDs, selective estrogen receptor degraders; CT, chemotherapy.
