# Supplementary material for: Beyond Chemotherapy: Network Meta‐Analysis Reveals Optimal Neoadjuvant Strategies for Luminal Breast Cancer
Source: Cancer Med. 2026 Feb 13;15(2):e71648. doi: 10.1002/cam4.71648 (PMC12902795; doi:10.1002/cam4.71648)
Supplement: Supplementary file 9 — Table S7: League table showing comparative efficacy of breast conservation therapy. [file CAM4-15-e71648-s002.docx]

Supplementary Table 7. League table showing comparative efficacy of breast conservation therapy

| CDK4/6 inhibitors + ET | 0.96 (0.60,1.52) | 0.93 (0.57,1.52) | 0.65 (0.22,1.90) | 0.70 (0.42,1.15) | 0.62 (0.36,1.08) |
| --- | --- | --- | --- | --- | --- |
| 1.04 (0.66,1.66) | AIs | 0.97 (0.64,1.46) | 0.67 (0.26,1.79) | 0.73 (0.49,1.08) | 0.65 (0.47,0.90) |
| 1.08 (0.66,1.77) | 1.03 (0.69,1.55) | SERDs | 0.70 (0.24,2.00) | 0.75 (0.44,1.27) | 0.67 (0.40,1.12) |
| 1.44 (0.87,2.37) | 1.37 (0.93,2.04) | 1.33 (0.78,2.26) | TKIs + ET | 0.93 (0.32,2.65) | 0.90 (0.57,1.40) |
| 1.55 (0.53,4.55) | 1.48 (0.56,3.92) | 1.44 (0.50,4.13) | 1.08 (0.38,3.08) | Chemotherapy | 0.97 (0.35,2.70) |
| 1.60 (0.93,2.77) | 1.53 (1.11,2.13) | 1.49 (0.89,2.48) | 1.03 (0.37,2.89) | 1.12 (0.71,1.74) | Tamoxifen |

*ET, endocrine therapy; AIs, aromatase inhibitors; TKIs, tyrosine kinase inhibitors; SERDs, selective estrogen receptor degraders; CT, chemotherapy.
