# Supplementary material for: Beyond Chemotherapy: Network Meta‐Analysis Reveals Optimal Neoadjuvant Strategies for Luminal Breast Cancer
Source: Cancer Med. 2026 Feb 13;15(2):e71648. doi: 10.1002/cam4.71648 (PMC12902795; doi:10.1002/cam4.71648)
Supplement: Supplementary file 10 — Table S8: League table showing comparative efficacy of completion rate. [file CAM4-15-e71648-s006.docx]

Supplementary Table 8. League table showing comparative efficacy of completion rate

| SERDs | 1.32 (0.30,5.83) | 1.56 (0.40,6.12) | 1.75 (0.70,4.34) | 1.82 (0.87,3.82) | 6.76 (2.36,19.35) |
| --- | --- | --- | --- | --- | --- |
| 0.76 (0.17,3.36) | CDK4/6 inhibitors + ET | 1.19 (0.66,2.15) | 1.33 (0.33,5.36) | 1.38 (0.38,5.04) | 5.14 (1.15,22.85) |
| 0.64 (0.16,2.51) | 0.84 (0.47,1.52) | Chemotherapy | 1.12 (0.32,3.96) | 1.17 (0.37,3.68) | 4.32 (1.10,17.03) |
| 0.57 (0.23,1.42) | 0.75 (0.19,3.04) | 0.89 (0.25,3.17) | Tamoxifen | 1.04 (0.61,1.77) | 3.87 (1.55,9.67) |
| 0.55 (0.26,1.15) | 0.72 (0.20,2.63) | 0.86 (0.27,2.70) | 0.96 (0.57,1.63) | AIs | 3.71 (1.75,7.84) |
| 0.15 (0.05,0.42) | 0.19 (0.04,0.87) | 0.23 (0.06,0.91) | 0.26 (0.10,0.65) | 0.27 (0.13,0.57) | TKIs + ET |

*ET, endocrine therapy; AIs, aromatase inhibitors; TKIs, tyrosine kinase inhibitors; SERDs, selective estrogen receptor degraders; CT, chemotherapy.
