# Supplementary material for: Beyond Chemotherapy: Network Meta‐Analysis Reveals Optimal Neoadjuvant Strategies for Luminal Breast Cancer
Source: Cancer Med. 2026 Feb 13;15(2):e71648. doi: 10.1002/cam4.71648 (PMC12902795; doi:10.1002/cam4.71648)
Supplement: Supplementary file 11 — Table S9: League table showing comparative efficacy of less adverse events (≥ grade 3). [file CAM4-15-e71648-s007.docx]

Supplementary Table 9. League table showing comparative efficacy of less adverse events (≥ grade 3).

| SERDs | 1.27 (0.09,18.00) | 2.56 (0.85,7.67) | 13.86 (4.13,46.53) | 29.39 (8.29,104.18) | 97.24 (9.34,1012.20) |
| --- | --- | --- | --- | --- | --- |
| 0.79 (0.06,11.19) | Tamoxifen | 2.02 (0.18,22.58) | 10.93 (0.93,129.03) | 23.17 (1.91,281.16) | 76.67 (3.19,1844.91) |
| 0.39 (0.13,1.17) | 0.50 (0.04,5.55) | AIs | 5.41 (3.24,9.04) | 11.49 (6.12,21.56) | 38.00 (4.80,301.06) |
| 0.07 (0.02,0.24) | 0.09 (0.01,1.08) | 0.18 (0.11,0.31) | CDK4/6 inhibitors + ET | 2.12 (1.28,3.52) | 7.02 (0.83,59.19) |
| 0.03 (0.01,0.12) | 0.04 (0.00,0.52) | 0.09 (0.05,0.16) | 0.47 (0.28,0.78) | TKIs + ET | 3.31 (0.38,28.79) |
| 0.01 (0.00,0.11) | 0.01 (0.00,0.31) | 0.03 (0.00,0.21) | 0.14 (0.02,1.20) | 0.30 (0.03,2.63) | Chemotherapy |

*ET, endocrine therapy; AIs, aromatase inhibitors; TKIs, tyrosine kinase inhibitors; SERDs, selective estrogen receptor degraders; CT, chemotherapy.
